# Socioeconomic impact and sufficiency of government financial support during COVID-19 pandemic: A retrospective study

**DOI:** 10.1371/journal.pone.0302979

**Published:** 2024-05-23

**Authors:** Wee Yeap Lau, Guek Nee Ke, Tien Ming Yip, Rachel Mei Ming Wong, Khalil Anwar Kamal, Shen Ching Lee, Stephen Carter, Rozainee Khairudin, Dasha Grajfoner

**Affiliations:** 1 Department of Decision Science, Faculty of Business and Economics, Universiti Malaya, Kuala Lumpur, Malaysia; 2 Department of Psychology, School of Social Sciences, Heriot-Watt University Malaysia, Putrajaya, Malaysia; 3 Malaysian Institute of Economic Research (MIER), Kuala Lumpur, Malaysia; 4 Edinburgh Business School, Heriot-Watt University Edinburgh, Scotland, United Kingdom; 5 National University of Malaysia, Bangi, Malaysia; 6 DOBA Business School, Maribor, Slovenia; Universiti Malaysia Sabah, MALAYSIA

## Abstract

This study examines the socioeconomic impact of the COVID-19 pandemic and the sufficiency of government support. Based on an online survey with 920 respondents, the cross-tabulation and binary logistic regression results show: firstly, in terms of loss of income, male respondents are more likely to have a loss of income as compared to female counterparts, and secondly, among different categories of employment status, the self-employed respondents are the most vulnerable group, given that more than 20 percent of them experienced loss of income due to the COVID-19 pandemic. Moreover, respondents working in small-and-medium enterprises (SMEs) and the informal sector are more likely to face loss of income as compared to respondents working in other sectors of employment. Likewise, respondents without tertiary education level are more likely to have a loss of income as compared to respondents with university certification. The baseline results highlight the insufficiency of government financial support programs based on the perspective of Malaysians from different demographic backgrounds. As a policy implication, the findings could guide the State in formulating the right policies for target groups who need more assistance than others in the community.

## Introduction

Coronavirus disease 2019 (COVID-19) is an illness caused by a novel coronavirus, namely severe acute respiratory syndrome coronavirus 2 (SARS-CoV-2; originally called 2019-nCoV) and was first detected in Wuhan City, Hubei Province, China, during an outbreak of respiratory illness. It was reported to the World Health Organization (WHO) for the first time on 31 December 2019. The WHO labelled the COVID-19 outbreak a worldwide health emergency on 30 January 2020. On 11 March 2020, COVID-19 was declared as a global pandemic by the WHO, the first time since 2009 when H1N1 influenza was proclaimed a pandemic [1]. The pandemic triggered a variety of effects on socioeconomic mechanisms, including labour markets, consumer behaviour, and global supply chains, all of which influenced the global economy. The financial markets were, and still are, undisputedly critical components of these mechanisms [2]. In the United States, a survey found that COVID-19 led to job losses among university students [3].

Moreover, the study found that students from low-income households were more vulnerable to the pandemic crisis, given that they were 55 percent more likely to be affected by COVID-19 as compared to their high-income peers. Meanwhile, a study proposed a household-level model to simulate the socioeconomic impact of COVID-19 on household consumption, saving and poverty rate [4]. The model found that in the absence of social protection from the government, the pandemic crisis would have had a massive economic shock on households. In particular, the poverty rate would have increased by almost 8.8 percent, while household consumption and saving would have dropped significantly. Likewise, similar findings were observed for developing countries. A survey found that residents from a rural village in Sierra Leone experienced a reduction in their income level compared to the pre-COVID-19 period [5]. The surveys also revealed that residents were losing their jobs and having difficulty in sustaining their daily lives due to the pandemic. Consistent results were found in African countries [6] and Nepal [7]. Overall, the existing findings suggested that COVID-19 would have a bearing on the income level, employment status and savings of economic agents. The coronavirus outbreak caused significant concerns about public health worldwide due to its rapid spread across the globe.

Subsequently, economic consequences were also brought to the forefront as more countries were switching to a work-from-home mandate to slow the spread of the virus, restricting travel, and shutting down schools [8]. Hence, according to the OECD, the new COVID-19 pandemic had severe economic consequences; potentially posing the biggest threat to the world economy [9]. While studies have been done on the economic effects of the pandemic, most papers have focused on the economic effects caused by pandemic-related deaths [10–15]. For example, when examining the SARS pandemic, [16] researched the pandemic’s health cost effects on China, Hong Kong, and Taiwan. In the case of Malaysia, the COVID-19 pandemic was not the first pandemic to hit the country. Before it, there was the SARS in 2002, the H1N1 flu pandemic in 2009 and the hand, foot, and mouth disease outbreak in 2018. However, while these three pandemics have impacted the economy, none of them had as widespread an effect as is currently seen with the COVID-19 pandemic. As the number of COVID-19 cases increased in Malaysia, the Malaysian Government began imposing movement restrictions to contain the spread of the virus. These actions resulted in an economic lockdown whereby economic activity was halted. Subsequently, the lockdown resulted in less consumer demand which thus affected consumption and caused a drop in sales. As sales suffered, the profit of listed companies dropped due to lower cash flow. The drop in profits affected their stock price. Thus, it is believed that there was a need to determine the impact of COVID-19 and the subsequent government responses to it on the Malaysian stock market.

## Literature review

Empirical studies have examined the impact of COVID-19 on a country’s macroeconomic performance, such as economic growth [17], stock market performance [18] and labour market conditions [19]. However, another strand of study investigated the impact of COVID-19 from the perspective of microeconomics [3–7]. Specifically, this study discussed the potential impact of COVID-19 on the socioeconomic wellbeing of economic agents, namely income, saving and employment status. This was important as it could inform policymakers on which group of people is more vulnerable to the pandemic crisis. Subsequently, a more focused policy could be implemented to compensate for the socioeconomic losses of economic agents as the result of COVID-19. Given that the focus of this study is on the socioeconomic impact of COVID-19, a review on the respective study was conducted.

In Malaysia, the COVID-19 pandemic crisis has seriously affected the labour market. Specifically, job losses in the country have increased by 42 percent year-on-year for the first quarter of 2020 [20]. Furthermore, the official figures from the Department of Statistics, Malaysia, show that the unemployment rate in April increased to 5 percent, as the number of people out of jobs went up to 778800, or 48.8 percent, as compared to the previous year [21]. However, the official reports and figures do not provide information on which group(s) of Malaysians are having a loss of income, loss of jobs and reduction in personal savings as the result of this pandemic. Specifically, there is no insight into which group(s) of Malaysians are most vulnerable to the COVID-19 pandemic crisis. This is important as it could provide input to the government in drafting their recovery policy so that no Malaysian is left behind. Motivated by the limitation, this study aims to investigate the impact of the COVID-19 pandemic on the socioeconomic wellbeing of Malaysians. Specifically, this study aims to reveal which group(s) of Malaysians are vulnerable to this pandemic from the perspective of income, savings, and job loss.

Aside from the psychological impact of COVID-19 on vulnerable groups such as older adults and women [22] The impact of COVID-19 on the psychological wellbeing of workers in Malaysia was a point of concern [23–26]. In their first study, the authors found that the relationship between psychological states and psychological wellbeing was successfully mediated by coping self-efficacy and resilience. The workers needed to have positive emotion, resilience, and coping efficacy during the pandemic period. The severity of COVID-19 on mental health in such a long-term health crisis cannot be ignored by the community and health authorities [23, 24].

In the follow-up study, investigations on the impact of the COVID-19 pandemic on employment-related issues, followed by different response strategies, were conducted [25]. With retrenchment measures being a common response strategy during economic downturns, many individuals faced unemployment. This study explored the effect of the COVID-19 pandemic-related retrenchment on the psychological wellbeing of the Malaysian workforce.

A purposive sample of 30 retrenched participants was interviewed via telephone during Malaysia’s Movement Control Order (MCO). Thematic analysis was subsequently conducted. Six themes emerged from the thematic analysis: (1) Retrenchment leading to the deterioration of psychological wellbeing; (2) Unemployment, financial strain, and economic uncertainty; (3) Emotions related to the COVID-19 virus; (4) Coping with lifestyle change; (5) Recommendations to improve the psychological wellbeing and mental health of the retrenched workforce, and (6) Career and financial support at the recovery phase. These two studies highlighted the link between the pandemic and psychological effects, and one suggested way to alleviate the hardship was through financial aid from the State or Federal Government. Hence, this study will examine the Impact of Government aid on the citizens.

### Government responses to COVID-19

**Fig 1:**
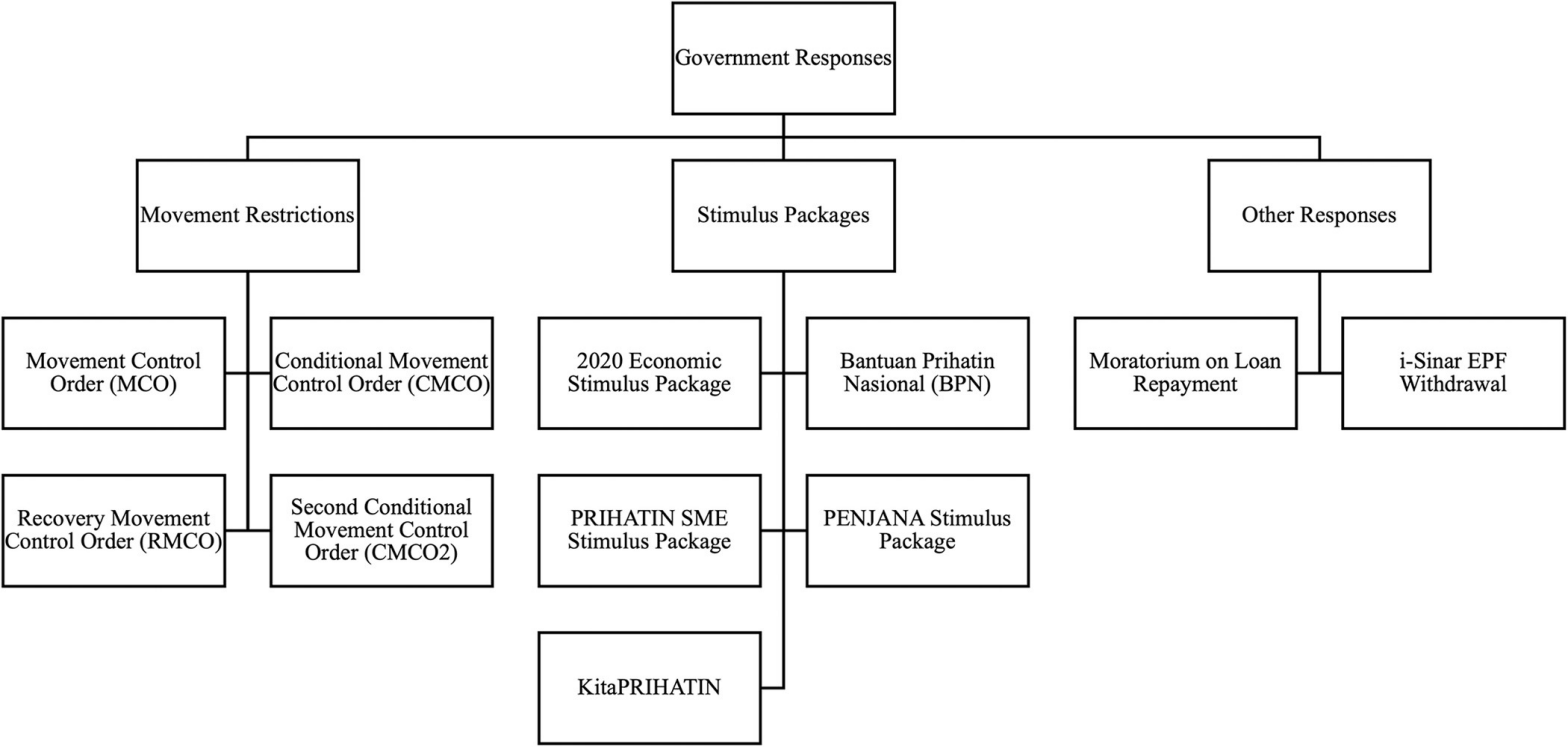
Malaysian government’s responses to COVID-19. *Note*: Author’s construction. The information in the above is sourced from various official reports and press releases.

As the number of cases increased, the Malaysian Government began imposing movement restrictions to prevent the spread of COVID-19 that resulted from social contact. Many activities were halted. The restrictions known as the Movement Control Order (MCO) had resulted in lesser consumer demand, and a drop in consumption and sales. Companies faced lower cash flow and drops in profit. In response to the pandemic and negative spill over to the livelihood of people, the government immediately executed several quick responses, including stimulus packages and other initiatives such as allowing employees in the private sectors to withdraw their monies from the Employee Provident Fund (EPF). In addition, the government also advised the commercial banks to impose a moratorium on loan repayment. In other words, borrowers would have additional time to repay their loans without being classified Non-Performing Loans (NPLs) by the commercial banks. Fig 1 shows the various government responses to COVID-19 throughout 2020.

As mentioned above, the Movement Control Order (MCO) was implemented from 18 March 2020 to 3 May 2020, though the MCO and was initially planned for only 2 weeks, from March 2020 until 31 March 2020.

At the end of the MCO, the government relaxed restrictions and implemented the Conditional Movement Control Order (CMCO) from 4 May 2020 to 9 June 2020, followed by the Recovery Movement Control Order (RMCO) from 10 June 2020 onwards, which further relaxed restrictions. Under RMCO, the government allowed non-essential economic activity to restart. Due to increased cases in Selangor and Kuala Lumpur, a Second Conditional Movement Control Order (CMCO2), tightening restrictions, was implemented in those states from 14 October 2020 to 12 January 2021.

Various stimulus packages were announced throughout the year, such as the 2020 Economic Stimulus Package, Bantuan Prihatin Nasional, PRIHATIN SME Economic Stimulus Package, PENJANA Stimulus Package and the KitaPRIHATIN Stimulus Package with a cumulative worth of RM305 billion (based on the MYR-USD exchange rate on 31 December 2020, an amount equivalent to USD 76.25 billion).

On 27 March 2020, the Malaysian Government announced Bantuan Prihatin Nasional (BPN) worth RM230 billion (USD 57.5 billion). The stimulus package was the most extensive stimulus package announced in 2020. Amongst the many highlights, this included an RM500 million (USD 125 million) allocation to the Health Ministry to fight the spread of COVID-19, a one-off cash assistance of RM1,600 to households earning below RM4,000, and an allocation of RM4.5 billion (USD 1.1 billion) prepared to aid small-and-medium enterprises (SMEs) and micro-businesses.

On 6 April 2020, the Malaysian Government announced an additional PRIHATIN SME Economic Stimulus Package worth RM10 billion (USD 2.5 billion), explicitly targeting SMEs. This stimulus package included two additional components to enhance the previously announced BPN. The two components were an additional RM7.9 billion added to the wage subsidy program announced in the BPN and a Special Prihatin Grant worth RM2.1 billion allocated to all eligible SMEs and micro-businesses.

In addition, on 5 June 2020, the Government announced the PENJANA Stimulus Package worth RM35 billion (USD 8.8 billion), which targeted households. The package included benefits for parents, including increased tax exemptions, e-vouchers for childcare services, and one-off cash handouts. Besides that, 1 GB of a free internet data plan was given to all users per day for educational and video conferencing purposes, RM50 was given for e-wallet usage, RM50 million (USD 12.5 million) was allocated for gig economies, and RM50 (USD 12.5 million) million was allocated to the B40 group for healthcare purposes.

On 23 September 2020, the Government announced the KitaPRIHATIN Stimulus Package worth RM10 billion (USD 2.5 billion). It was a stimulus package that targeted SMEs and was introduced due to feedback that many SMEs and micro-businesses were not eligible for the benefits in the BPN due to being unregistered with the Social Security Organisation (SOCSO) before 01 April 2020. This additional stimulus package was estimated to benefit 1.3 million workers. In addition, a Special Prihatin Grant was also introduced to aid micro-businesses further. Besides that, the Government also approved EPF withdrawals via i-Sinar and granted a moratorium on loan repayment.

## Data and methodology

Prior to data collection, ethical approval was obtained by the Ethics Committee at School of Social Sciences, Heriot-Watt University (2020-0461-1466) on 8^th^ June 2020. The sample size was estimated using a single population proportion formula based on Malaysia’s estimated population size of 32.7 million at the time of study, [n = (z^2^ *p (1- p)/ d2] [27, 28] by considering the total percentage of 53.2% from the distribution of income categories from B40 and M40, a 95% confidence interval for this estimate (z = 1.96), and a 5% margin of error (d = 0.05). A minimum sample size of 383 people was required for the survey. According to the Household Income Estimate and Incidence of Poverty Report in 2019, the income distribution of income categories T20, M40 and B40 was at 46.8%, 37.2% and 16.0%, respectively [29].

Data collection took place between 24^th^ June to 19^th^ July 2020, and participants were presented the information sheet and required to provide informed consent digitally before answering the online questionnaire. Any participant who did not provide informed consent were unable to proceed with answering the questionnaire. As all participants were above the age of 18, parental or guardian consent was not required. No personally identifying information was captured, and responses were anonymised using participant IDs.

A total of 1,217 responses were collected, but those that did not provide sufficient data on the measures of interest were considered invalid and excluded from analysis and participants who did not complete the survey were removed entirely. After considering this criterion, 297 responses were excluded from the analysis, leaving a final sample size of 920, which exceeded the recommended size. Participants completed the survey during the implementation of Malaysia’s Movement Control Order (MCO).

For this study, a bivariate analysis was used to examine the socioeconomic impact of COVID-19 on Malaysians with different socio-demographic characteristics. In particular, the questions on loss of income, loss of job and a reduction in personal savings are the dependent variables and were tabulated by selected socio-demographic variables, namely gender, type of employment, sector of employment, education level, marital status, and place of residence.

This provided a first indication on which group(s) of Malaysians are more likely to be affected by COVID-19 based on the three major aspects. Next, as many of the independent variables are inter-correlated and have confounding effects on the dependent variables, binary logistic regression analyses, which allow for multivariate analysis, were conducted to ensure the robustness of the baseline results. Utilizing binary logistic regression, it was possible to show the odds of Malaysians having loss of income, loss of job and a reduction in personal saving due to the COVID-19 pandemic crisis.

Table 1 shows the list of variables used in this study. Based on the existing studies as mentioned earlier, this study defined the socioeconomic impact of COVID-19 pandemic as loss of income, job loss and a reduction in personal savings. These three dependent variables were numerated by a categorical scale, with 0 equal to ‘no’ and 1 equal to ‘yes’. The three dependent variables were selected based on the past studies [3–7], whereby these studies highlighted the economic impact of COVID-19 pandemic from the perspective of individual’s income level, employment status and saving. Moreover, from the perspective of macroeconomics, the three dependent variables measure the overall health and stability of the economy, thereby providing useful information to guide the conduct of fiscal policy. On the other hand, the selected demographic variables were gender, employment status, sector of employment, education level, marital status, and place of residence.

**Table 1:** List of variables.

Dependent variable	Label
Loss of income	0 = No, 1 = Yes
Loss of job	0 = No, 1 = Yes
Reduce in personal saving	0 = No, 1 = Yes
**Independent variables**	
Gender	0 = Female, 1 = Male
Employment Status	0 = Permanent
	1 = Self-employed
	2 = Unemployed
	3 = Contract
Sector of employment	0 = Government
	1 = Listed company
	2 = Private company (Unlisted)
	3 = Small and Medium Enterprises (SMEs)
	4 = Informal sector
Education level	0 = With university certificate
	1 = Without university certificate
Marital Status	0 = Single, 1 = Married
Place of residence	0 = Rural, 1 = Urban

## Discussion

### Demographic information

Table 2 presents the summary distribution of respondents based on selected socio-demographic characteristics. As observed, more than 50 percent of the respondents had a permanent job, whilst self-employed and unemployed respondents comprised 13.7 and 12.7 percent respectively of the total sample. Likewise, 54.2 percent of the respondents worked in private companies, while less than 10 percent of them were civil servants (6.6 percent). Furthermore, more than half of the respondents (59.3 percent) were educated to a tertiary level. Most of the respondents were currently resident in an urban area (84.6 percent).

**Table 2:** Percentage distribution of respondent by demographic characteristics.

Demographic characteristics	Sample size	Percentage
**Gender**		
Female	501	54.5
Male	419	45.5
**Employment status**		
Permanent	611	66.4
Self-employed	126	13.7
Unemployed	117	12.7
Contract	66	7.2
**Sector of employment**		
Government	127	6.6
Listed company	113	12.3
Private company (Unlisted)	499	54.2
Small and Medium Enterprises (SME)	103	11.2
Informal sector	78	6.5
**Education level**		
With university certificate	546	59.3
Without university certificate	374	40.7
**Marital status**		
Single	489	53.2
Married	431	46.8
**Place of residence**		
Rural	142	15.4
Urban	778	84.6

### Baseline results: Socioeconomic impact

Table 3 shows the result for the cross tabulation. By focusing on the loss of income, as observed, male respondents were more likely to have a loss of income as compared to female counterparts. The percentage distribution was 11 and 5.6 percent respectively. Furthermore, the chi-square test of association was significant at the 1 percent level, indicating that there was an association between loss of income and gender. Based on the results, male respondents were found to be affected the most in terms of income as compared to female respondents.

**Table 3:** Percentage distribution of impact of COVID-19 by selected demographic characteristics.

	Dependent variables					
	Loss of income		Loss of job		Reduction in saving	
	No	Yes	No	Yes	No	Yes
**Gender**						
Female	94.4	5.6	96.8	3.2	88.6	11.4
Male	89.0	11.0	95.2	4.8	85.0	15.0
Pearson Chi-Square statistics	8.96**		1.51		2.69	
**Employment status**						
Permanent	94.1	5.9	98.2	1.8	89.0	11.0
Self-employed	77.8	22.2	95.2	4.8	77.0	23.0
Unemployed	94.9	5.1	91.5	8.5	88.0	12.0
Contract	93.9	6.1	86.4	13.6	84.8	15.2
Pearson Chi-Square statistics	39.77***		30.77***		13.75***	
**Sector of employment**						
Government	98.4	1.6	98.4	1.6	85.0	15.0
Listed companies	95.6	4.4	97.3	2.7	92.0	8.0
Private companies (Unlisted)	91.6	8.4	95.0	5.0	88.4	11.6
Small and Medium Enterprises (SME)	83.5	16.5	96.1	3.9	75.7	24.3
Informal sector	89.7	10.3	97.4	2.6	88.5	11.5
Pearson Chi-Square statistics	19.77***		4.29		15.47***	
**Education level**						
With university certificate	94.1	5.9	96.5	3.5	88.6	13.4
Without university certificate	88.8	11.2	95.5	4.5	87.4	12.6
Pearson Chi-Square statistics	8.65***		0.67		0.12	
**Marital status**						
Single	91.8	8.2	95.3	4.7	86.5	13.5
Married	92.1	7.9	97.0	3.0	87.5	12.5
Pearson Chi-Square statistics	0.02		1.73		0.18	
**Place of residence**						
Rural	90.1	9.9	97.2	2.8	90.8	9.2
Urban	92.3	7.7	95.9	4.1	86.2	13.8
Pearson Chi-Square statistics	0.74		0.53		2.23	

Note

***p<0.01

**p<0.05

*p<0.1

Next, among different categories of employment status, there was a higher proportion of self-employed respondents who experienced a loss of income (22.2 percent) as compared to respondents with permanent job (5.9 percent), unemployed respondents (5.1 percent) and respondents with contract- based jobs (6.1 percent). Likewise, the chi-square test was significant at the 1 percent level, indicating that there were differences in the distribution of loss of income among respondents with different employment status.

For the sector of employment, more than 10 percent of the respondents from SMEs and informal sector were experiencing a loss of income. The percentage distribution was 16.5 and 10.3 percent, respectively. The percentage distribution was relatively higher than respondents from the government sector, listed and private companies. The chi-square test was significant at the 1 percent, suggesting that there was a relationship between loss of income and sector of employment. Based on the results, it was expected that respondents from SMEs and the informal sector were more likely to have a reduction in their income as compared to other sectors of employment.

Among respondents with different education levels, results indicated that respondents without a tertiary education qualification were more likely to have a loss of income (11.2 percent) as compared to those with university certification (5.9 percent). Lastly, the chi-square test of association was insignificant for marital status and place of residence, indicating that the proportion of loss of income was about the same for respondents who were currently single or married and in rural or urban area.

For job loss, the chi-square test of association was significant only for employment status. The proportion of job loss was higher for respondents with contract- based jobs (13.6 percent), unemployed respondents (8.5 percent) and self-employed respondents (4.8 percent) as compared to respondents with a permanent job (1.8 percent).

For reduction in personal savings, similarly, results showed that respondents with contract- based jobs (15.2 percent), unemployed respondents (12 percent) and self-employed respondents (23 percent) were more likely to have a reduction in their personal savings as compared to respondents with a permanent job (11 percent). Given that respondents with a permanent job are less likely to have a loss of income and job as compared to respondents with other employment status, it can be expected that the COVID-19 pandemic had less influence on their saving level.

Among the sector of employment, more than 20 percent of the respondents who were working in SMEs were experiencing a reduction in their personal savings as the result of the COVID-19 pandemic (24.3 percent). The percentage distribution was almost twice as high than respondents working in the government sector, private company, and informal sectors.

### Robustness check: Socioeconomic impact

Table 4 shows the binary logistic regression for the three dependent variables. By focusing on the loss of income, notably, the results were in line with the above bivariate analysis, whereby the odds of having loss of income among female respondents were 0.5 times less than their male counterparts. This indicated that male respondents are the more vulnerable group and more likely to have a loss of income as compared to female respondents.

**Table 4:** Binary logistic regression.

		Dependent variables		
		Loss of income	Loss of job	Reduction in savings
Independent variables	n	Exp (B)	Exp (B)	Exp (B)
**Gender**				
Female	501	0.531**	0.652	0.802
Male (Reference)	419	1.0	1.0	1.0
**Employment status**		(0.002)***	(0.000)***	(0.064)*
Self-employed	126	3.108***	2.585*	2.096***
Unemployed	117	0.907	6.456***	1.051
Contract	66	0.933	7.600***	1.367
Permanent (Reference)	611	1.0	1.0	1.0
**Sector of employment**		(0.069)*	(0.347)	(0.070)*
Listed companies	113	3.076	2.900	0.489*
Private companies (unlisted)	499	5.202**	4.128*	0.687
Small and Medium Enterprises (SME)	103	7.749***	2.860	1.377
Informal sector	78	7.106***	1.861	0.734
Government (Reference)	127	1.0	1.0	1.0
**Education level**				
Without university certificate	374	1.616*	1.286	0.890
With university certificate (Reference)	546	1.0	1.0	1.0
**Marital status**				
Married	431	0.714	0.631	0.838
Single (Reference)	489	1.0	1.0	1.0
**Place of residence**				
Urban	778	0.799	1.493	1.609
Rural (Reference)	142	1.0	1.0	1.0
Constant		0.021	0.005	0.138

Note

***p<0.01

**p<0.05

*p<0.1

Among different employment status, the odds of having a loss of income among self-employed respondents was 3 times higher than respondents with a permanent job. Moreover, the odds for self-employed respondents were the highest among the four employment statuses. The results were in line with the above bivariate analysis, in which self-employed respondents were the most vulnerable group and they were more likely to have a loss of income as compared to respondents with other employment status.

For the sector of employment, notably, the results were consistent with the bivariate analysis. As such, the odds of having a loss of income among respondents working in a private company, SMEs and informal sector were 5, 8 and 7 times, respectively, greater than respondents working in the government sector. Similarly, respondents without university certification were almost 2 times more likely to have a loss of income as compared to those with university certification.

Furthermore, all the variables mentioned above were significant, indicating that gender, employment status, sector of employment and education level were important in explaining the loss of income among Malaysians during the COVID-19 pandemic. By and large, both cross tabulation and binary logistic regression showed consistent results.

For job loss, consistent with the above bivariate analysis, the odds of losing a job for self-employed respondents, unemployed respondents, and respondents with contract- based jobs were 2, 5 and 7 times higher, respectively, than respondents with a permanent job.

For reduction in personal savings, the binary logistic result showed that the odds of losing personal saving among self-employed respondents was 2 times higher than respondents with a permanent job. This was in line with the earlier results in loss of income and job loss. As such, self-employed respondents were found to be more likely to have a loss of income and job as compared to respondents with a permanent job. In such situations, self-employed respondents had to utilize their own savings to sustain their daily lives. Hence, this led to a reduction in their personal savings during the pandemic crisis. Meanwhile, respondents with a permanent job were less likely to be affected by the COVID-19 pandemic in terms of income and job, therefore it could be expected that they could sustain their daily life without using their private savings.

The marginal effects for each covariate were estimated to complement the odds ratio, and the results were tabulated in Table 5. By and large, the results were consistent with the odds ratio estimated from the binary logistic regression. Specifically, the female’s probability of having loss of income would be 4.3 percentage points lower than male counterparts. For the employment status, the probability of self-employed respondents to loss their income was 10.4 percentage point higher than respondents with a permanent job. Moreover, respondents working in a private company, SMEs and informal sector exhibited a higher probability of having loss of income than respondents working in the government sector. For education, respondents without education certificate were found to have a higher probability of having loss of income as compared to their counterparts with education certificate.

**Table 5:** Marginal effects estimated from binary logistic regression.

		Dependent variables		
		Loss of income	Loss of job	Reduction in savings
Independent variables	n	Average marginal effects		
**Gender**				
Female	501	-0.043**	-0.015	-0.024
Male (Reference)	419			
**Employment status**				
Self-employed	126	0.104***	0.027	0.096**
Unemployed	117	-0.005	0.085***	0.005
Contract	66	-0.004	0.101***	0.035
Permanent (Reference)	611			
**Sector of employment**				
Listed companies	113	0.035	0.023	-0.074*
Private companies (unlisted)	499	0.067***	0.037***	-0.044
Small and Medium Enterprises (SME)	103	0.101***	0.023	0.047
Informal sector	78	0.092**	0.011	-0.037
Government (Reference)	127			
**Education level**				
Without university certificate	374	0.033*	0.009	-0.013
With university certificate (Reference)	546			
**Marital status**				
Married	431	-0.023	-0.016	-0.019
Single (Reference)	489			
**Place of residence**				
Urban	778	-0.015	0.014	0.052
Rural (Reference)	142			

Note

***p<0.01

**p<0.05

*p<0.1

The above marginal effects are estimated using the MARGIN STATA command.

For job loss, consistent with the above bivariate analysis, the probability of losing a job for unemployed respondents and respondents with contract- based jobs were higher than respondents with a permanent job. For sector of employment, respondents working in private companies were reported to have a higher probability of losing their job.

For reduction in personal savings, the probability of losing personal saving among self-employed respondents was 9.6 percentage points higher than respondents with a permanent job. Conversely, respondents working in listed companies were found to have a lower probability of having reduction in saving as compared to respondents working in the public sector.

In conclusion, the bivariate and multivariate analyses provided insights on which segments of Malaysian citizens are vulnerable to the COVID-19 pandemic. The results found that male respondents, respondents without a permanent job, respondents working in non-public sector and respondents without education certificate have exhibited substantial economic losses, in the form of income, job and savings, as a result of the COVID-19 pandemic.

### Results: Sufficiency of government financial support

**Fig 2:**
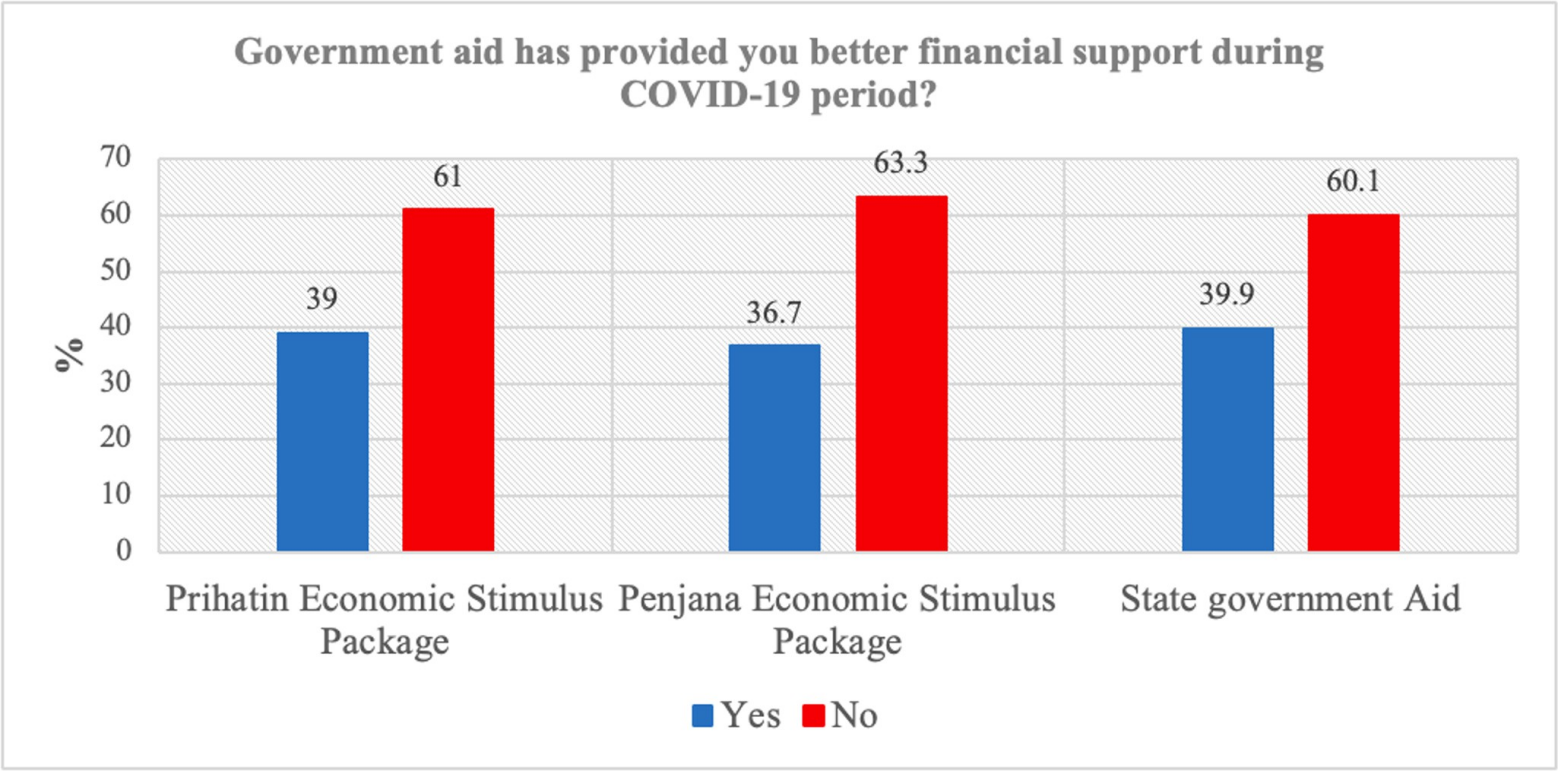
Percentage distribution of respondent by government financial support.

Fig 2 shows the basic distribution of respondents’ attitudes towards the sufficiency of government financial support programs. Overall, respondents opined that the three government financial support programs were insufficient in providing support to Malaysians during the COVID-19 period.

### Baseline results: Sufficiency of government financial support

Table 6 shows the result for the cross tabulation. By focusing on the Prihatin Economic Stimulus Package, as observed, the chi-square test of association was significant for sector of employment, education level, and marital status. This indicated that there were differences in the distribution of opinion toward the sufficiency of Prihatin Economic Stimulus Package among respondents with different sector of employment, education level and marital status.

**Table 6:** Percentage distribution of government financial support by selected demographic characteristics.

	Government financial support					
Demographic variables	Prihatin		Penjana		State Government Aid	
	No	Yes	No	Yes	No	Yes
**Gender**						
Female	60.1	39.9	62.3	37.7	63.2	36.8
Male	62.1	37.9	64.1	35.9	57.5	42.5
Pearson Chi-Square statistics	0.373		0.311		3.158*	
**Employment status**						
Permanent	61.4	38.6	64.0	36.0	60.4	39.6
Self-employed	55.6	44.4	58.7	41.3	56.3	43.7
Unemployed	62.4	37.6	65.0	35.0	63.2	36.8
Contract	65.2	34.8	62.1	37.9	59.1	40.9
Pearson Chi-Square statistics	2.179		1.436		1.273	
**Sector of employment**						
Government	49.6	50.4	51.2	48.8	44.1	55.9
Listed companies	70.8	29.2	69.0	31.0	69.9	30.1
Private companies (Unlisted)	61.3	38.7	63.9	36.1	59.7	40.3
Small and Medium Enterprises (SME)	59.2	40.8	67.0	33.0	68.0	32.0
Informal sector	65.4	34.6	65.4	34.6	64.1	35.9
Pearson Chi-Square statistics	12.275**		10.453**		21.311***	
**Education level**						
With university certificate	63.2	36.8	65.8	34.2	66.7	33.3
Without university certificate	57.8	42.2	59.6	40.4	50.5	49.5
Pearson Chi-Square statistics	2.753*		3.583*		24.090***	
**Marital status**						
Single	56.0	44.0	59.3	40.7	55.0	45.0
Married	66.6	33.4	67.7	32.3	65.9	34.1
Pearson Chi-Square statistics	10.729***		7.029***		11.316***	
**Place of residence**						
Rural	60.6	39.4	59.2	40.8	50.7	49.3
Urban	61.1	38.9	64.0	36.0	61.8	38.2
Pearson Chi-Square statistics	0.012		1.218		6.194**	

Note

***p<0.01

**p<0.05

*p<0.1

For sector of employment, it can be observed that respondents working in the non-government sector were more likely to view that the Prihatin Economic Stimulus Package was insufficient in providing financial support to them during the COVID-19 period. For education level, surprisingly, respondents with university certification were more likely to say that the financial support by government was insufficient to sustain their life during COVID-19 period as compared to respondents without university certification. The percentage distribution was 63.2 and 57.8 percent, respectively. Furthermore, there was a higher proportion of married couples who opined that the financial support by government was insufficient to cover expenses as compared to respondents who were single. The percentage distribution was 66.6 and 56.0 percent, respectively. Notably, similar findings could be observed for the Penjana Economic Stimulus Package and State Government Aid.

### Robustness check: Sufficiency of government financial support

Table 7 shows the binary logistic regression for the three dependent variables. Notably, the estimation results were consistent with the above bivariate analysis, in which sector of employment, education level and marital status were found to have a significant impact on the dependent variables.

**Table 7:** Binary logistic regression: Sufficiency of government financial support.

		Dependent variables		
		Prihatin	Penjana	State government aid
Independent variables	n	Exp (B)	Exp (B)	Exp (B)
**Gender**				
Female	501	0.866	1.039	0.727
Male (Reference)	419	1.0	1.0	1.0
**Employment status**		(0.229)	(0.414)	(0.247)
Self-employed	126	0.732	0.719	0.746
Unemployed	117	1.172	1.116	1.334
Contract	66	1.347	1.049	1.106
Permanent (Reference)	611	1.0	1.0	1.0
**Sector of employment**		(0.019)**	(0.039)**	(0.000)***
Listed companies	113	2.434***	2.038**	2.887***
Private companies (unlisted)	499	1.648**	1.718***	1.986***
Small and Medium Enterprises (SME)	103	1.593	2.102**	2.973***
Informal sector	78	2.031**	1.830**	2.601***
Government (Reference)	127	1.0	1.0	1.0
**Education level**				
Without university certificate	374	0.770*	0.773*	0.496***
With university certificate (Reference)	546	1.0	1.0	1.0
**Marital status**				
Married	431	1.652***	1.483***	1.729***
Single (Reference)	489	1.0	1.0	1.0
**Place of residence**				
Urban	778	0.915	1.122	1.271
Rural (Reference)	142	1.0	1.0	1.0
Constant		0.995	0.879	0.762

Note

***p<0.01

**p<0.05

*p<0.1

For sector of employment, it can be observed that the odds of opining that the Prihatin Economic Stimulus Package, Penjana Economic Stimulus Package and State Government Aid were insufficient in providing financial support to Malaysian was higher for respondents working in the non-government sector. Likewise, respondents with university certification were found to have a higher probability in arguing that the financial support by government was insufficient to cover their expenses as compared to respondents without university certification. Lastly, the odds of opining government financial supports were insufficient among married couple was about 2 times higher than respondents who were single. By and large, both cross tabulation and binary logistic regression showed consistent results.

Table 8 shows the marginal effect estimates for all the covariates. Notably, the estimation results were consistent with the above odds ratio. For sector of employment, it can be observed that the probability of opining that the Prihatin Economic Stimulus Package, Penjana Economic Stimulus Package and State Government Aid were insufficient in providing financial support to Malaysian was higher for respondents working in the non-government sector. Likewise, respondents without university certification were found to have a lower probability in arguing that the financial support by government was insufficient to cover their expenses as compared to respondents with university certification. Lastly, the probability of opining government financial supports was insufficient among married couples couple was higher than respondents who were single.

**Table 8:** Marginal effect estimated from binary logistic regression: Sufficiency of government financial support.

		Dependent variables		
		Prihatin	Penjana	State government aid
Independent variables	n	Average marginal effects		
**Gender**				
Female	501	-0.033	0.009	-0.071**
Male (Reference)	419			
**Employment status**				
Self-employed	126	-0.074	-0.077	-0.066
Unemployed	117	0.036	0.024	0.062
Contract	66	0.066	0.011	0.022
Permanent (Reference)	611			
**Sector of employment**				
Listed companies	113	0.206***	0.167***	0.242***
Private companies (unlisted)	499	0.120**	0.129***	0.161***
Small and Medium Enterprises (SME)	103	0.112*	0.174***	0.249***
Informal sector	78	0.168**	0.144**	0.221***
Government (Reference)	127			
**Education level**				
Without university certificate	374	-0.060*	-0.058*	-0.156***
With university certificate (Reference)	546			
**Marital status**				
Married	431	0.115***	0.089***	0.122***
Single (Reference)	489			
**Place of residence**				
Urban	778	-0.021	0.026	0.053
(Reference)	142			

Note

***p<0.01

**p<0.05

*p<0.1

The above marginal effects are estimated using the MARGIN STATA command.

In conclusion, the bivariate and multivariate analyses provided insights on which segments of Malaysian citizens are receiving insufficient financial support from the government. The results found that respondents working in the non-government sector, respondents with education certification and married couple have received insufficient financial support from the government during COVID-19 period.

## Conclusion and summary of key findings

Several observations can be noted from the results. As mentioned, this study consisted of two parts. The first part of study on the socioeconomic Impact of COVID-19 had the following findings: First, in terms of loss of income, male respondents were more likely to have a loss of income, compared to their female counterparts. Next, among different categories of employment status, a self-employed respondent was the most vulnerable group, given that more than 20 percent of them were having loss of income due to the COVID-19 pandemic. Moreover, respondents working in SMEs and the informal sector were more likely to face loss of income as compared to respondents working in other sectors of employment. Likewise, respondents without tertiary education, were more likely to have a loss of income as compared to respondents with university certification.

Second, in terms of job loss, respondents who were self-employed, unemployed and in contract- based jobs were more likely to have a job loss as compared to those with a permanent position. Third, in terms of personal savings, consistently, respondents with contract- based jobs, unemployed respondents and self-employed respondents were more likely to have a reduction in their personal saving as compared to respondents with a permanent job. Next, among sectors of employment, respondents working in SMEs was the most vulnerable group, given that more than 20 percent them were experiencing a reduction in their personal savings as the result of the COVID-19 pandemic. Overall, the binary logistic regression results were consistent with the baseline bivariate analysis. Hence, the results were robust and not affected by different estimation methods.

The second part of the study focused on the sufficiency of Government financial support and proffered the following findings: The baseline results highlighted the insufficiency of government financial support programs based on the perspective of Malaysians from different demographic backgrounds. Result showed that the government financial support programs, namely Prihatin Economic Stimulus Package, Penjana Economic Stimulus Package and State Government Aid were insufficient in providing financial support to Malaysians during the COVID-19 pandemic period. Respondents working in the non-government sector, respondents with university certification and married couples argued that the financial support given by government was insufficient for them to sustain their daily lives during the COVID-19 pandemic period.

In conclusion, the COVID-19 pandemic has been one of the toughest ordeals faced by human society historically. This study is important for shedding light on which segments of Malaysians are vulnerable to the COVID-19 pandemic and are receiving insufficient financial support from the government. Specifically, the male respondents, respondents without a permanent job, respondents working in non-public sector and respondents without education certificate were vulnerable to the COVID-19 pandemic. In addition, the respondents working in the non-government sector, respondents with education certification and married couples were found to receive insufficient financial support from the government during COVID-19 period. The findings provide useful input to guide the Malaysian government in formulating the right policies for target groups who need more assistance than others in the community.
